# The adipose tissue has an apical-basal polarity required for Col IV–dependent cell–cell adhesion

**DOI:** 10.1083/jcb.202504139

**Published:** 2026-01-06

**Authors:** Jameela Almasoud, Cyril Andrieu, Bren Hunyi Lee, Anna Franz

**Affiliations:** 1 Department of Cell and Developmental Biology, University College London, London, UK

## Abstract

In epithelia, the apical-basal polarity machinery positions E-cadherin–based adherens junctions at the apical-lateral border to mediate cell–cell adhesion. The *Drosophila* adipose tissue, the fat body, forms a monolayer in which integrin-binding to collagen IV intercellular concentrations mediates cell–cell adhesion. How these atypical adhesion complexes form is unknown. Here we show that the fat body has apical-basal polarity, with aPKC, Crumbs, and Par-6 on the opposite side of Lgl and Dlg. Collagen IV, Laminin, Perlecan, and Nidogen are abundant in the basal basement membrane, while collagen IV predominates in the apical basement membrane. Crumbs, aPKC, Scribble, and Lgl knockdown in the fat body lead to cell–cell adhesion defects. Moreover, aPKC is essential for the formation of collagen IV intercellular concentrations. We further show that during fat body remodeling, Ecdysone regulates the loss of apical-basal polarity and collagen IV intercellular concentrations to induce cell–cell dissociation and swimming migration. Our work hence uncovers a novel role for apical-basal polarity in the *Drosophila* adipose tissue in regulating cell–cell adhesion via collagen IV intercellular concentrations.

## Teaser

Collagen IV intercellular concentrations mediate cell–cell adhesion in the *Drosophila* adipose tissue. This study finds that the adipose tissue has apical-basal cell polarity, which regulates this adhesion. It further shows that during tissue remodeling, Ecdysone regulates the loss of apical-basal polarity and collagen IV intercellular concentrations to induce cell–cell dissociation and swimming migration.

## Introduction

Mesoderm-derived adipocytes form true tissues by tightly associating with each other. In mammals, the adipose tissue is distributed over multiple subcutaneous and visceral depots (Pope et al., 2016). In *Drosophila* larvae, adipocytes are large polyploid cells that are organized in a single continuous monolayer, called the fat body. This tissue lies inside the body cavity and is surrounded by hemolymph, the body fluid. The fat body tissue bifurcates at the anterior end of the animal into two sheets that extend on each side toward the posterior end of the animal, surrounding the internal organs, including the gut, like a bilateral apron. The mechanisms that maintain adipose tissue architecture and its functional significance remain largely elusive. In contrast, the tissue architecture of epithelia and its function have been extensively studied. In *Drosophila*, cell–cell adhesion through E-cadherin–based adherens junctions at the apical-lateral border mediates the formation of sheets, which is dictated by apical-basal cell polarity (Coopman and Djiane, 2016). A conserved set of polarity proteins determines the various domains in epithelial cells, as uncovered mostly through studies in *Drosophila* and *Caenorhabditis elegans*. These showed that the apical domain is specified by the transmembrane protein Crumbs, the adaptor protein Stardust, and the Par-6/atypical protein kinase C (aPKC) complex. Bazooka (Baz in flies, named Par-3 in other organisms) defines the boundary between the apical and lateral domains. It plays a key role in positioning the apical adherens junctions and localizing the apical factors, which then exclude Baz from the apical domain through aPKC-dependent phosphorylation of Baz (Franz and Riechmann, 2010; Harris and Peifer, 2005; Krahn et al., 2010; Morais-de-Sa et al., 2010; Nunes de Almeida et al., 2019; Walther and Pichaud, 2010). Discs large (Dlg), Lethal (2) giant larvae (Lgl), and Scribble (Scrib) mark the rest of the lateral domain and the basal domain (Assemat et al., 2008). Mutual antagonism between apical and lateral factors then ensures the maintenance of the identity of the apical and lateral domains (Bilder et al., 2003; Tanentzapf and Tepass, 2003). Moreover, a basement membrane (BM) composed of the extracellular matrix proteins collagen IV, Perlecan, Nidogen, and Laminin underlies the basal domain of epithelial cells (Hynes, 2012).

In contrast to epithelia, the fat body is not known to have an apical-basal cell polarity. Cell–cell adhesion here has been proposed to be mediated by two alternative mechanisms, via E-cadherin–based adherens junctions (Jia et al., 2014) or via collagen IV intercellular concentrations (CIVICs) (Dai et al., 2017). In agreement with a role of E-cadherin in fat body cell (FBC)–cell adhesion, it was reported that E-cadherin is localized at cell–cell vertices in the larval fat body, and this localization is then lost during fat body remodeling (FBR) as cells dissociate (Jia et al., 2014). More recently, it was shown that extracellular collagen IV–containing punctae are found spread along the cell–cell vertices in the pericellular space of the larval fat body (Dai et al., 2017). Neighboring cells attach to these CIVICs via integrin and Syndecan receptors, which is essential for cell–cell adhesion (Dai et al., 2017). However, it remains unknown how CIVICs form in the pericellular space between neighboring FBCs.

In embryonic development and disease, epithelia can undergo an epithelial-to-mesenchymal transition (EMT). During this process, epithelial cells lose their apical-basal cell polarity as well as cell–cell and cell–BM adhesion to gain mesenchymal characteristics, enabling them to migrate (Thiery et al., 2009). Some cancer cells can also undergo an epithelial-to-amoeboid transition (EAT) and use amoeboid cell migration to leave the tumor (Graziani et al., 2022).

The *Drosophila* fat body undergoes FBR during metamorphosis at the early pupal stage at 4–14 h after puparium formation (APF). Cells lose cell–cell and cell–BM adhesion and become individual cells that spread across the body within the hemolymph following head eversion (Bond et al., 2011; Nelliot et al., 2006). This process is induced by signaling through the steroid hormone Ecdysone and requires expression of the matrix metalloproteinases MMP1 and MMP2 (Bond et al., 2011; Jia et al., 2014). We recently discovered that at a later pupal stage, at 16 h APF, FBCs in the pupa are not passively floating in hemolymph but are instead motile. They use swimming migration, an unusual subtype of amoeboid cell migration, to respond to wounds (Franz et al., 2018; Martin et al., 2020) and to patrol the pupa (Andrieu et al., 2025). This suggests that FBCs must become migratory following FBR.

Overall, the larval fat body appears to have some similarities to epithelia. Both form cell layers through cell–cell and cell–BM adhesion. Yet cells in the fat body have a BM on each surface (Brac, 1983), while epithelia have a BM underlying only the basal surface (Khalilgharibi and Mao, 2021). Moreover, the cells in the fat body adhere to each other via CIVICs and are not known to have an apical-basal polarity. This raises the question of how CIVIC formation in the fat body is regulated and whether the apical-basal polarity machinery is involved.

Here we examine the epithelial apical-basal cell polarity network in the larval fat body and show that this tissue displays an apical-basal cell polarity. We also show that integrin is enriched near the basal BM, which contains abundant amounts of collagen IV, Laminin, Perlecan, and Nidogen. In contrast, less integrin is found near the apical BM containing predominantly collagen IV. We find that aPKC, which regulates the localization of Crumbs, Baz, and Dlg, is essential for cell–cell adhesion by mediating CIVIC formation in the larval fat body. We further show that apical-basal cell polarity and CIVICs are lost early during FBR, which is regulated by Ecdysone signaling.

## Results

### The larval fat body tissue exhibits apical-basal cell polarity

**Figure S1.**
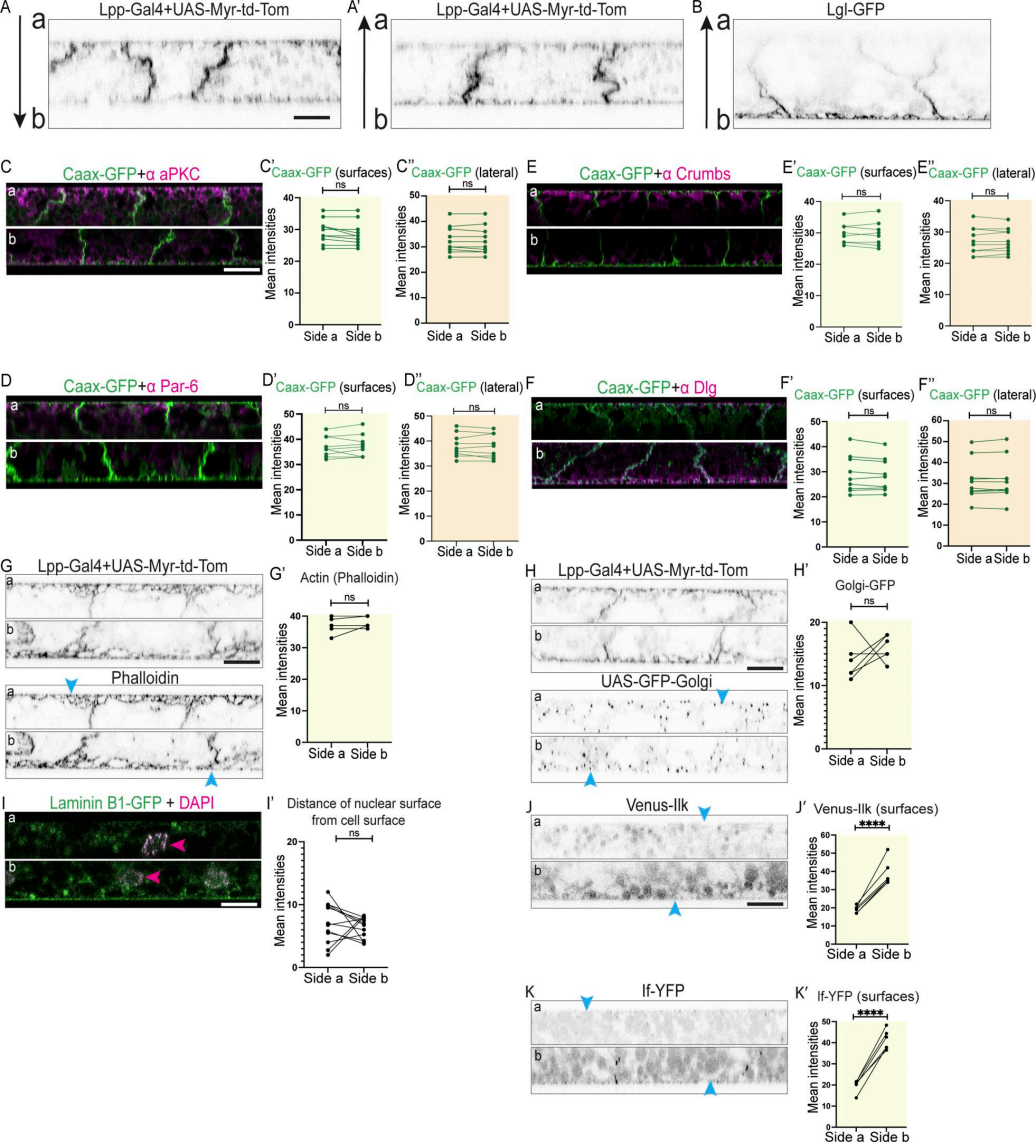
**The larval fat body tissue exhibits apical-basal cell polarity. (A and B)** Confocal images of larval fat bodies expressing Lpp-Gal4+UAS-Myr-td-Tom (A) or Lgl-GFP (B; imaged across the whole tissue from side a [top] toward side b [bottom] [A] or from side b [bottom] toward side a [top] [A′ and B] shown in lateral view, arrow indicates direction of imaging). **(C–F″)** Confocal images of CAAX-GFP–expressing larval fat body immunostained for aPKC (C), Par-6 (D), Crumbs (E), and Dlg (F; imaged separately starting from side a [top] or b [bottom], merged channels shown in lateral view, CAAX-GFP in green and antibody stain in magenta). Quantification of mean intensities of CAAX-GFP on surface ROIs (‘, yellow background) or lateral ROIs (‘‘, orange background) on sides a and b (mean of mean intensities from several ROIs at the surface or lateral domain of same tissue, data paired by tissue, quantifications using same samples as in Fig. 1, B–E; *n*: 10 tissues, 3 surface or lateral ROIs per side (Fig. 1, B′–E′ and B″–E″). Paired two-sided *t* test, ns P > 0.05. Related to Fig. 1, B–E. **(G–I′)** Confocal images of larval fat bodies expressing Lpp-Gal4+UAS-Myr-td-Tom with Phalloidin-488 staining to label actin (G) or expressing Lpp-Gal4+UAS-Myr-td-Tom+UAS-GFP-Golgi (H) or expressing Laminin-B1-GFP with DAPI staining (I), imaged on sides a (top) and b (bottom), shown in lateral view. Quantification of mean intensities of Phalloidin (G′) or GFP-Golgi (H′; in ROI proximal to the cell surfaces, paired for each fat body tissue, *n*: six tissues, three surface ROIs per tissue per side) or of distance of nuclear surface from cell surface (I′, paired for each fat body tissue, *n*: six tissues, three images per side). Paired two-sided *t* test, ns P > 0.05. **(J–K′)** Confocal images of Venus-Ilk–expressing (J) or If-YFP–expressing (K) larval fat body (side a [top] and b [bottom], lateral view). Quantification of mean intensity of Venus-Ilk (J′) and If-YFP (K′) on surfaces on sides a and b (mean of mean intensities from several ROIs, data paired by tissue; *n*: six tissues, three surface ROIs per side). Unpaired two-sided *t* test, ****P < 0.0001. Related to Fig. 2. Scale bars: 20 µm (A–K).

**Figure 1.**
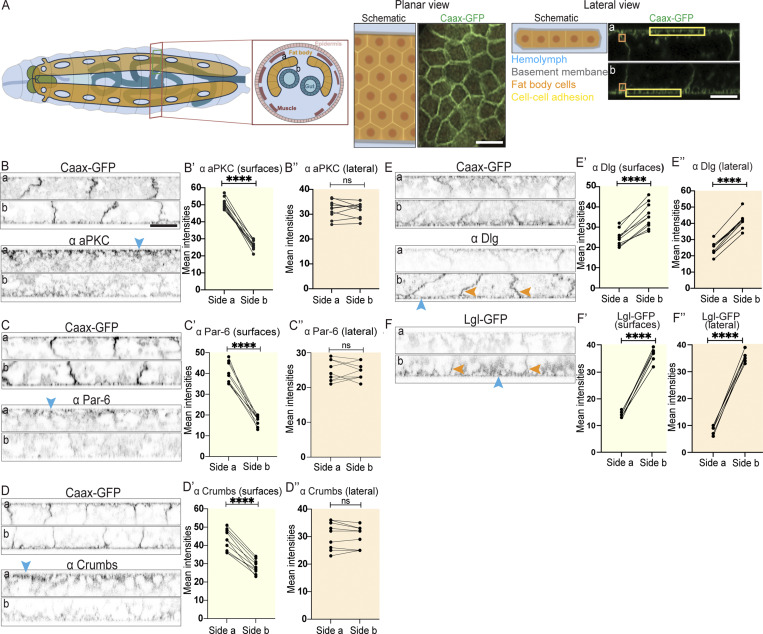
**The larval fat body tissue exhibits apical-basal cell polarity. (A)** Schematic of wandering third instar larva (dorsal view and cross section on left) showing location of fat body (orange, sides a and b of fat body shown in cross section) in relation to brain (green), digestive system (blue), muscle (dark red), and epidermis (pink). Schematic and confocal images of CAAX-GFP–expressing fat body in planar view and lateral view (imaged from both sides, showing yellow surface ROIs and orange lateral ROIs used for intensity quantifications). **(B–F″)** Confocal images of CAAX-GFP–expressing (B–E) or Lgl-GFP–expressing (F) larval fat body immunostained for aPKC (B), Par-6 (C), Crumbs (D), and Dlg (E; imaged from side a [top] and b [bottom], shown in lateral view, with blue and orange arrowheads pointing at cell surfaces or lateral domains, respectively). Quantification of mean intensities of aPKC (B′ and B″), Par-6 (C′ and C″), Crumbs (D′ and D″), Dlg (E′ and E″), and Lgl-GFP (F′ and F″) on surface ROIs (‘, yellow background) or lateral ROIs (‘‘, orange background) on sides a and b (mean of mean intensities from several ROIs at the surface or lateral domain of the same tissue, data paired by tissue; *n*: 10 tissues, 3 surface or lateral ROIs per side [B′–E′ and B″–E″] and *n*: six tissues, two surface or lateral ROIs per side [F′ and F″]). Paired two-sided *t* test, ****P < 0.0001, ns P > 0.05. Scale bars: 50 µm (A—planar view image), 20 µm (A—lateral view image and B–F).

To establish whether the fat body tissue in wandering third instar stage larvae has an apical-basal cell polarity, we performed antibody stainings for a range of classic polarity proteins known to localize to the apical (aPKC, Par-6, and Crumbs) or basolateral domain (Dlg) in classic epithelia. We found that it was not an optimal approach to compare intensities at both cell surfaces by imaging FBCs from top to bottom at a high resolution due to the large size of these cuboidal cells and light scattering issues (Fig. S1, A and B). Hence, we mounted the fat body between two coverslips and imaged both sides of the tissue separately. To distinguish the two sides of the fat body (side [a] facing outward toward the body wall and side [b] facing inward toward the gut, Fig. 1 A on the left), we took advantage of a morphological asymmetry noticeable in the larval fat body tissue architecture and only used the right sheet of the fat body for our experiments (see Materials and methods for more details). In addition to the immunostaining for particular polarity proteins, CAAX-GFP expression was used to visualize membranes to find the cell surface and lateral domains. We then quantified the mean intensities for CAAX-GFP and the antibody stain in several ROIs at the surface as well as at the lateral domain near the surface (shown in yellow and orange boxes, respectively, in Fig. 1 A on the right) on each side of the fat body for each animal (data in graphs paired for each tissue). These quantifications showed that while CAAX-GFP was always equally distributed on both surfaces and lateral domains (Fig. S1, C–F″), aPKC, Par-6, and Crumbs consistently localized more strongly to the surface of side (a) with no difference in the lateral domains (Fig. 1, B–D″). In contrast, Dlg localized more strongly to the surface on the opposite side, side (b), as well as to the lateral domain near side (b) (Fig. 1, E and E″). A similar localization was also observed for another basolateral protein, Lgl, using an Lgl-GFP protein trap line (Fig. 1, F–F″ and Fig. S1 B). In contrast to this cellular polarity, there was no obvious apical-basal asymmetry in the actin network, the Golgi apparatus, and nuclear positioning (Fig. S1, G–I′).

Altogether, these results suggest that the larval fat body displays an apical-basal polarity with aPKC, Par-6, and Crumbs found on side (a), henceforth referred to as the apical side, and Dlg as well as Lgl on side (b), referred to as the basal side, as well as spread along the basolateral domain.

### The larval fat body has distinct apical and basal BMs

**Figure 2.**
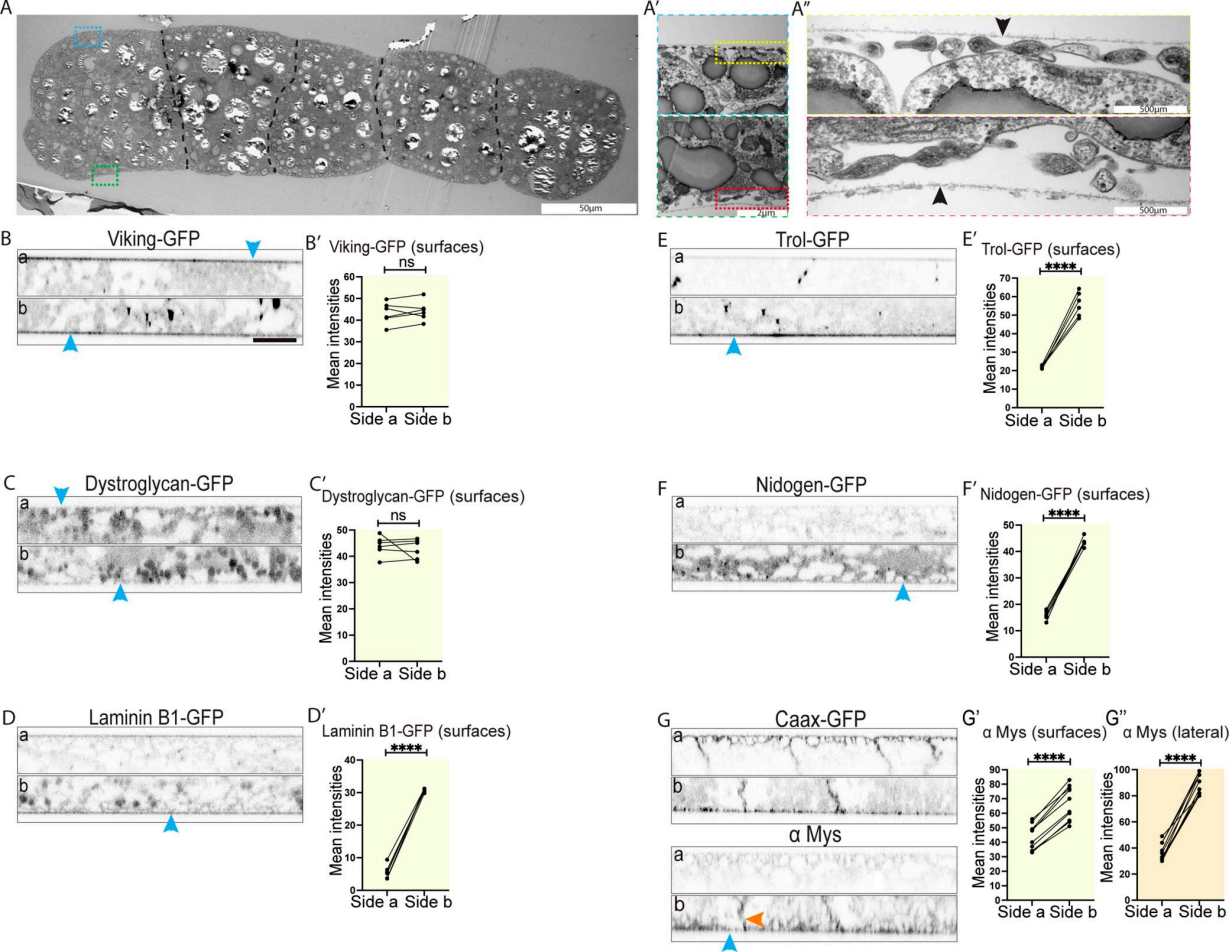
**The larval fat body has distinct apical and basal BMs. (A–A″)** Transmission electron microscopy images at different magnifications of WT larval fat body showing the BM (black arrowheads) near the cell surface on opposite sides of the tissue. Note the presence of microridge protrusions at the cell surface (A″). **(B–F′)** Confocal images of Viking-GFP–expressing (B), Dystroglycan-GFP–expressing (C), Laminin B1-GFP–expressing (D), Trol (Perlecan)-GFP–expressing (E), or Nidogen-GFP–expressing (F) larval fat body (side a [top] and b [bottom], lateral view, blue arrowheads pointing at cell surface). Quantification of mean intensity of Viking-GFP (B′), Dystroglycan-GFP (C′), Laminin B1-GFP (D′), Trol-GFP (E′), or Nidogen-GFP (F′) on surfaces on side a and b (mean intensities from several ROIs, data paired by tissue; *n*: six tissues, three surface ROIs per side). Unpaired two-sided *t* test, ****P < 0.0001, ns P > 0.05. **(G–G″)** Confocal images of CAAX-GFP–expressing larval fat body immunostained for Mys. (G, side a [top] and b [bottom], lateral view, blue and orange arrowheads pointing at cell surface or lateral domain, respectively). Quantification of mean intensity of Mys on surfaces (‘, yellow) or lateral domain (‘’, orange) on sides a and b (mean of mean intensities from several ROIs, data paired by tissue; *n*: 10 tissues, 6 surface ROIs [G′] or 10 lateral ROIs [G″] per side). Paired two-sided *t* test, ****P < 0.0001. Scale bars: 50 µm (A), 2 µm (A′), 500 nm (A″), and 20 µm (B–G).

The fat body is known to have BMs on both surfaces, which are thought to have the same composition (Brac, 1983). Having discovered an apicobasal polarity in this tissue, we wondered whether the apical and basal BMs might be different. Electron microscopy of dissected fat body did not reveal any obvious differences between the basal and apical BMs (Fig. 2, A–A″). Next, we looked at the localization of all the major ECM components as well as at components of the integrin and Dystroglyan cell–ECM adhesion complexes, mostly using fluorescent protein trap lines. We found that Viking-GFP (Col IV α2 chain-GFP) and Dystroglycan-GFP were found at similar levels at both surfaces of the fat body (Fig. 2, B–C′). In contrast, Laminin-B1-GFP, Trol-GFP (Perlecan-GFP), and Nidogen-GFP were all strongly enriched on the basal surface (Fig. 2, D–F′), similar to Mys (integrin βPS), If-YFP (integrin αPS2), and Venus–integrin-linked kinase (Ilk), three components of the integrin complex (Fig. 2, G and G′; and Fig. S1, J–K′). In addition, Mys was also more concentrated at the basolateral than the apicolateral domain of the fat body (Fig. 2 G″), similar to CIVIC distribution. Together, this suggests that the larval fat body has two distinct BMs. All the major ECM proteins, Laminin, Nidogen, Perlecan, and collagen IV, as well as the integrin complex, are abundantly present at the basal BM. In contrast, collagen IV is the predominant ECM protein found in the apical BM where less integrin is present.

### E-cadherin RNAi is not sufficient to cause cell–cell adhesion defects in the larval fat body

**Figure 3.**
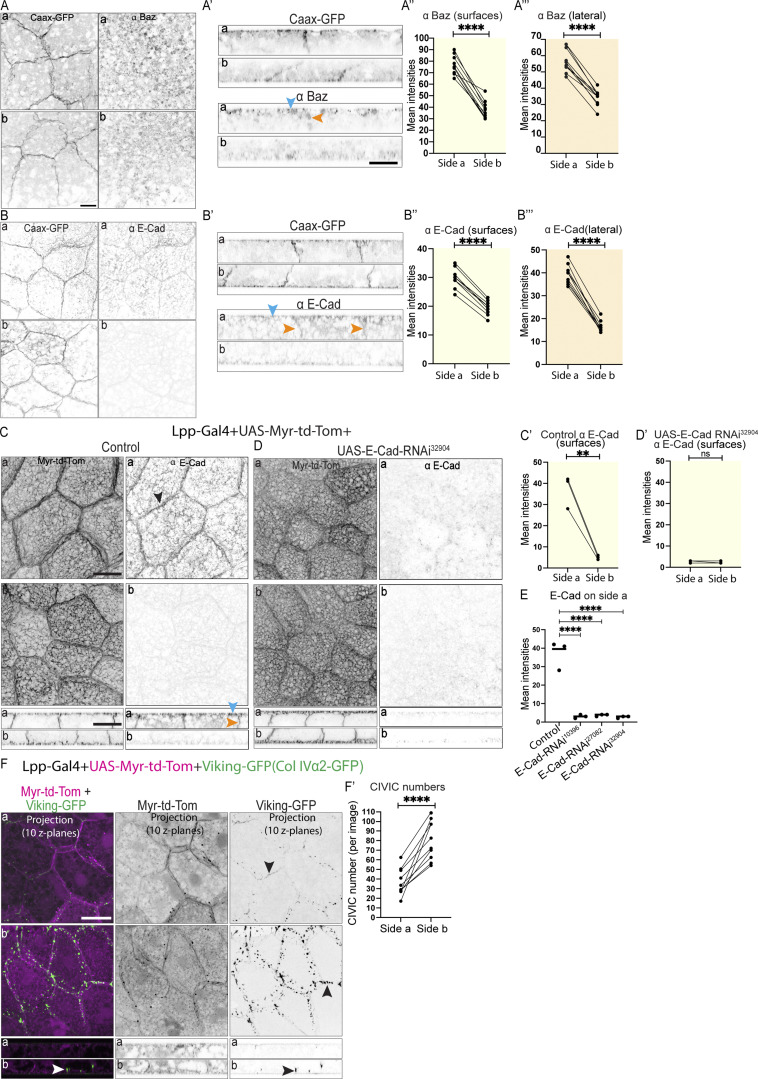
**E-cadherin RNAi is not sufficient to cause cell–cell adhesion defects in the larval fat body. (A–B‴)** Confocal images of CAAX-GFP–expressing larval fat body immunostained for Baz (A and A′), and E-cadherin (B, B′; side a [top] and b [bottom] in planar view [A and B] and lateral view [A′ and B′], blue and orange arrowheads pointing at cell surfaces or lateral domains, respectively). Quantification of mean intensities of Baz (A″ and A‴) and E-cadherin (B′ and B‴) on surfaces (‘, yellow) or lateral domains (‘’, orange) on side a and b (mean of mean intensities from several ROIs, data paired by tissue; *n*: 10 tissues, 10 or 5 surface or lateral ROIs per side for Baz or E–Cadherin, respectively [A″–B″ and A‴–B‴]). Paired two-sided *t* test, ****P < 0.0001. **(C–E)** Confocal images of larval fat body expressing Lpp-Gal4+UAS-Myr-td-Tomato +control (C) or +UAS-E-cadherin RNAi^32904^ (D) immunostained for E-cadherin (side a and b shown in planar [top] and lateral views [bottom], black arrowhead pointing at cell–cell vertex, blue and orange arrowheads pointing at surface or lateral domain, respectively). Note that the same control images are displayed in Fig. 3 C and Fig. S2 A. Fig. 3, C–E and Fig. S2, A–C′ are the results from the same experiment and hence the control is the same for both. Quantification of mean intensity of E-cadherin for control (C′) or UAS-E-cadherin RNAi^32904^ (D′) on surfaces on sides a and b (mean of mean intensities from several ROIs, data paired by tissue; *n*: three tissues, three surface ROIs per side). Unpaired two-sided *t* test, ****P < 0.0001. Quantification of mean intensities of E-cadherin for control, UAS-E-cadherin RNAi^32904^ (from C′ and D′), UAS-E-cadherin RNAi^103962^, and UAS-E-cadherin RNAi^27082^ (from Fig. S2, B′ and C′) shown for side a (E). Ordinary one-way multiple comparisons ANOVA, ****P < 0.0001. **(F and F′)** Confocal images of larval fat body expressing Lpp-Gal4+UAS-Myr-td-Tomato+Viking-GFP (F; side a and b shown in planar [top] and lateral views [bottom], black and white arrowheads pointing at CIVICs at cell–cell vertices; merge and single channels of Z projection of 10 Z planes at 2.5–5 μm from cell surface). Quantification of CIVIC numbers per image (F′, using thresholded Z projection images of 10 Z planes of Viking-GFP channel [2.5–5 μm from cell surface], *n*: 10 tissues, 10 Z projection images per tissue and side, data paired by tissue). Paired two-sided *t* test, ****P < 0.0001, ns P > 0.05. Scale bars: 20 µm (A–D and F).

Having discovered that the larval fat body tissue displays an apical-basal cell polarity, we wondered whether this polarity is involved in the regulation of cell–cell adhesion in the fat body, as in epithelia. Cell–cell adhesion in the fat body has been suggested to involve E-cadherin–based adherens junctions (Jia et al., 2014). However, whether E-cadherin is essential for cell–cell adhesion in the larval fat body is not known. To investigate the role of E-cadherin in cell–cell adhesion in the larval fat body further, we assessed the localization of E-cadherin and Baz, which are both known to localize to adherens junctions in the form of an apicolateral belt in many epithelia in *Drosophila* (Harris and Peifer, 2005; Morais-de-Sa et al., 2010; Walther and Pichaud, 2010). Our immunostainings using Baz and E-cadherin antibodies overall resulted in a rather diffuse signal that labeled cell–cell vertices (marked with either CAAX-GFP or Lpp-Gal4+UAS-Myr-td-Tom), albeit relatively weakly, and showed no clear belt-like apicolateral concentration (Fig. 3, A–B′). Our intensity quantifications revealed that both Baz and E-Cad localized more strongly to the apical surface and apicolateral domain than to the basal and basolateral domain, respectively (Fig. 3, A′–A‴ and B′–B‴).

**Figure S2.**
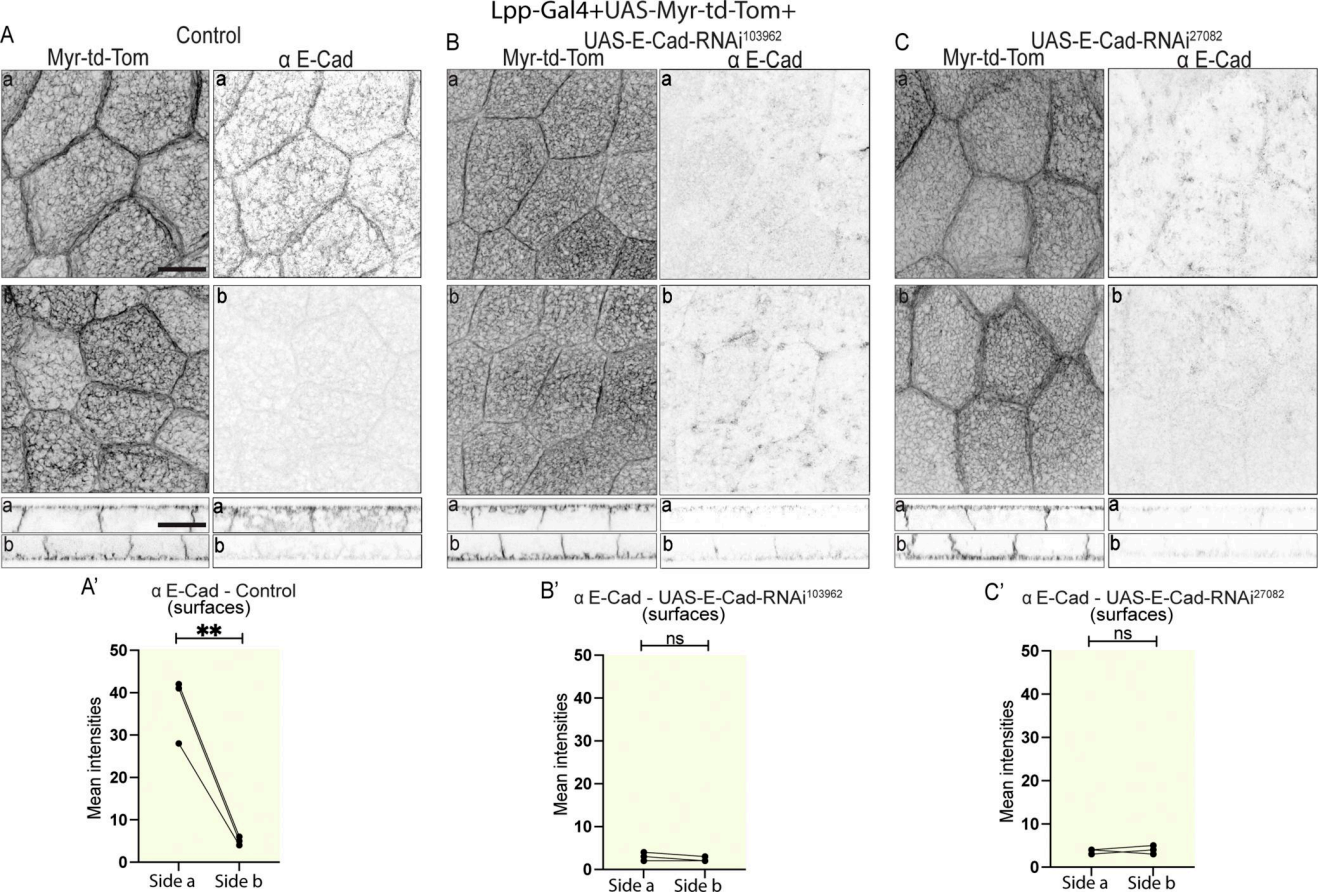
**E-cadherin RNAi is not sufficient to induce cell–cell dissociation in larval fat body. (A–C**′**)** Confocal images of larval fat body expressing Lpp-Gal4+UAS-Myr-td-Tomato +control (A), +UAS-E-cadherin RNAi^103962^ (B), or +UAS-E-cadherin RNAi^27082^ (C) immunostained for E-cadherin (sides a and b shown in planar [top] and lateral views [bottom]). Related to Fig. 3, C–E. Note that the same control images are displayed in Fig. 3 C and Fig. S2 A. Fig. 3, C–E and Fig. S2, A–C′ are the results from the same experiment and hence the control is the same for both. Quantification of mean intensity of E-cadherin for control (A′), UAS-E-cadherin RNAi^103962^ (B′), or UAS-E-cadherin RNAi^27082^ (C′) on surfaces on sides a and b (mean of mean intensities from several ROIs, data paired by tissue; *n*: three tissues, three surface ROIs per side). Unpaired two-sided *t* test, **P < 0.01, ns P > 0.05. Scale bars: 20 µm (A–C).

Next, we tested if E-cadherin knockdown in the larval fat body is sufficient to cause cell–cell dissociation. Lpp-Gal4 was used to drive expression of UAS-E-Cad RNAi together with a membrane marker (UAS-Myr-td-Tom) specifically in the fat body throughout larval stages. The third instar larval fat body was then immunostained for E-cadherin to assess knockdown efficiency. E-Cad RNAi using three different independent RNAi constructs did not result in any cell–cell dissociation despite resulting in a strong reduction in E-cadherin staining, demonstrating the efficiency of the RNAi knockdowns (Fig. 3, C–E and Fig. S2). This suggests that E-Cad knockdown is not sufficient to cause cell–cell dissociation in the larval fat body.

### Cell–cell adhesion in the larval fat body involves collagen IV intercellular concentrations

Apart from adherens junctions, integrin binding to pericellular CIVICs has been suggested to regulate cell–cell adhesion in the fat body (Dai et al., 2017). Col IVα1 RNAi, Col IVα2 RNAi, or integrin β RNAi causes moderate cell–cell dissociation of FBCs, particularly on tricellular vertices (Dai et al., 2017), strongly suggesting that CIVICs mediate cell–cell adhesion in the fat body. Hence, we decided to study the distribution of CIVICs in the larval fat body along the lateral domain by looking at Viking-GFP expressed under its endogenous promoter as well as Lpp-Gal4+UAS-Myr-td-Tom to visualize membranes. We then quantified the number of CIVICs in the lateral domain near the apical and basal surfaces (using Z projections of 10 Z-layers at 2.5–5 μm from the cell surface on side a or b). As reported before (Dai et al., 2017), we saw CIVICs as punctae spread along the cell–cell vertices of FBCs (Fig. 3 F, note that the broader distribution of CIVICs along cell–cell vertices Z projection is due to vertices sloping along the Z axis). However, while CIVICs were scattered along most of the lateral domain, we found fewer CIVICs present at the area of lateral domain near the apical surface (Fig. 3, F and F′ side a). Our data, together with the findings from a previous study (Dai et al., 2017), suggest that cell–cell adhesion in the larval fat body appears to be mainly mediated by CIVICs.

### Apical-basal polarity regulates collagen IV–dependent cell–cell adhesion in the larval fat body

**Figure 4.**
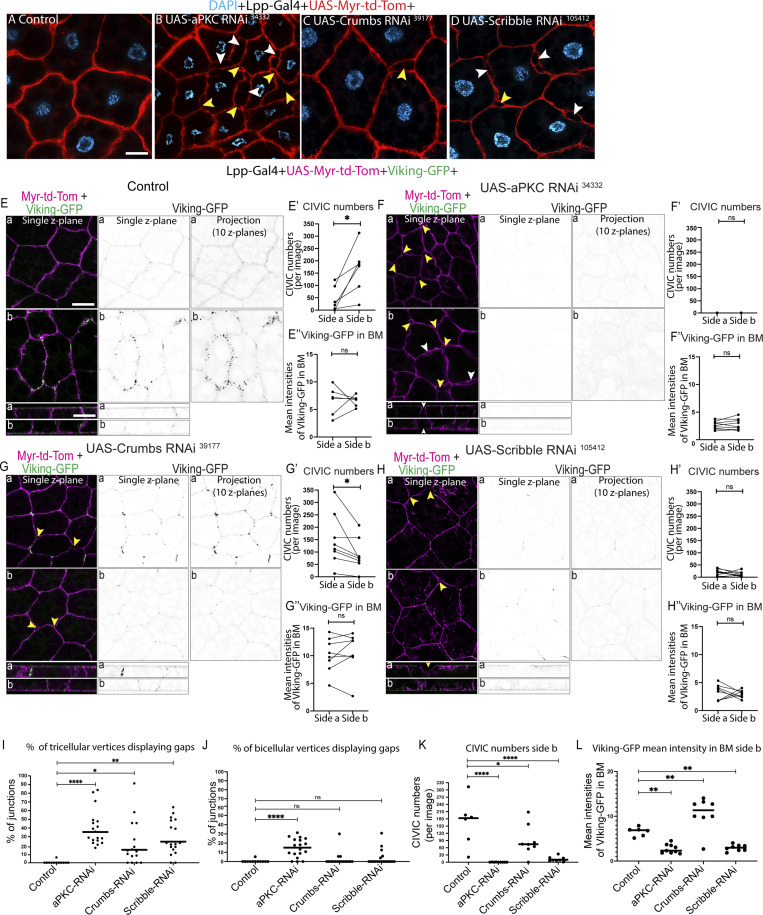
**Apical-basal polarity regulates collagen IV–dependent cell–cell adhesion in the larval fat body. (A–D)** Confocal single Z plane images of DAPI-stained, larval fat body expressing Lpp-Gal4+UAS-Myr-td-Tomato +control (A), UAS-aPKC RNAi^34332^ (B), UAS-Crumbs RNAi^39117^ (C), and UAS-Scribble RNAi^105412^ (D; yellow or white arrowheads showing gaps at tricellular or bicellular cell–cell vertices, respectively). **(E–L)** Confocal images of larval fat body expressing Lpp-Gal4+UAS-Myr-td-Tomato+Viking-GFP +control (E), +UAS-aPKC RNAi^34332^ (F), +UAS-Crumbs RNAi^39117^ (G), and +UAS-Scribble RNAi^105412^ (H; showing images of merged channels [single Z plane] and Viking-GFP channel [single Z plane and Z projection of 10 layers 2.5–5 μm from cell surface]; yellow or white arrow showing gaps at tricellular or bicellular vertices, respectively). Quantification of percentage of tricellular or bicellular cell–cell vertices containing gaps per image (I and J, respectively) from E–H (*n*: six images [control], nine images [UAS-aPKC RNAi^34332^], eight images [UAS-Crumbs RNAi^39117^], and eight images [UAS-Scribble RNAi^10541^], each from different animals). Kruskal–Wallis test followed by Dunn’s multiple comparisons, ****P < 0.0001, **P < 0.01, *P < 0.05, and ns P > 0.05. Quantification of mean CIVIC numbers (E′, F′, G′, and H′; from thresholded Z projection images of 10 Z planes of Viking-GFP channel [2.5–5 μm from cell surface], *n*: 10 tissues, 10 Z projection images per tissue and side, data paired by tissue) and mean intensity of Viking-GFP in the BM (E″, F″, G″, and H″; using surface ROIs in lateral view, data paired by tissue; *n*: six pupae [control], nine pupae [UAS-aPKC RNAi^34332^], eight pupae [UAS-Crumbs RNAi^39117^], and eight pupae [UAS-Scribble RNAi^105412^], 1 ROI per side for each tissue) on sides a and b. Paired two-sided *t* test, ****P < 0.0001. Quantification of CIVIC numbers (K) and mean intensity of Viking-GFP in the BM (L) for control, UAS-aPKC RNAi^34332^, UAS-Crumbs RNAi^39117^, and UAS-Scribble RNAi^105412^ on side b. Ordinary one-way multiple comparisons ANOVA, ****P < 0.0001, ***P < 0.001, **P < 0.01, *P < 0.05, and ns P > 0.05. Scale bars; 20 µm (A–D and E–H).

**Figure S3.**
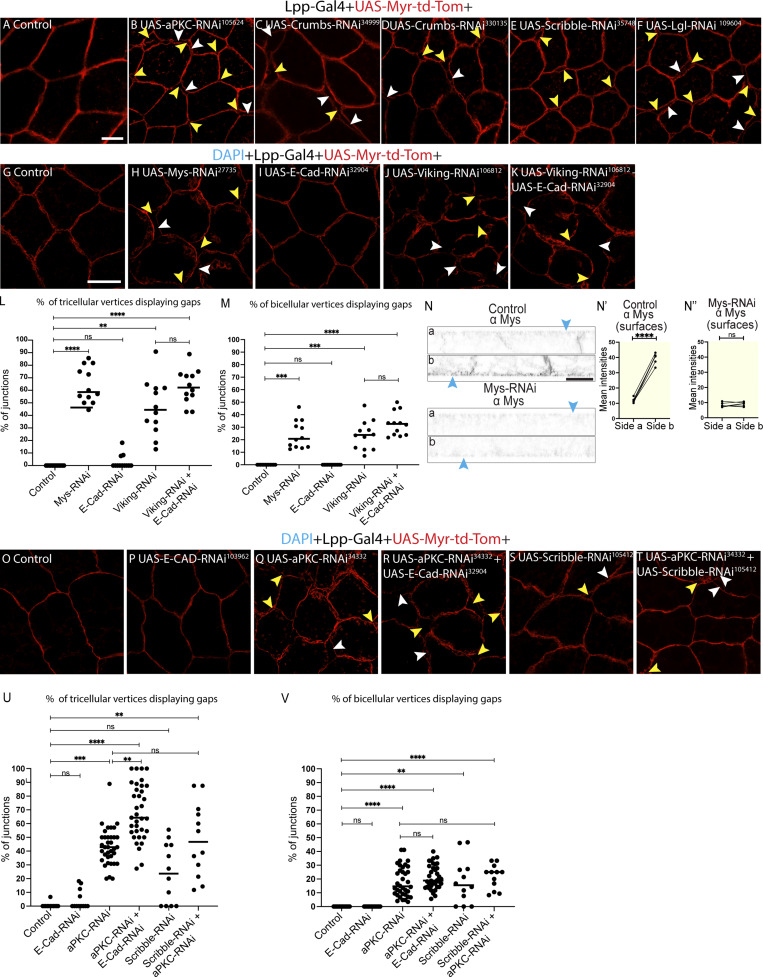
**Apical-basal cell polarity is needed for collagen-IV–dependent cell–cell adhesion—related to Fig. 2, A–D. (A–F)** Confocal single Z plane images of larval fat body expressing Lpp-Gal4+UAS-Myr-td-Tomato +control (A), UAS-aPKC RNAi^105624^ (B), UAS-Crumbs RNAi^34999^ (C), UAS-Crumbs RNAi^330135^ (D), UAS-Scribble RNAi^35748^ (E), or UAS-Lgl RNAi^109604^ (D; yellow or white arrow showing gaps at tricellular or bicellular vertices, respectively). **(G–N″)** Confocal single Z plane images of larval fat body expressing Lpp-Gal4+UAS-Myr-td-Tomato +control (G), UAS-Mys RNAi^27735^ (H), UAS-E-Cad RNAi^32904^ (I), UAS-Viking RNAi^106812^ (J), or UAS-Viking RNAi^106812^+UAS-E-Cad RNAi^32904^ (K; yellow or white arrow showing gaps at tricellular or bicellular vertices, respectively). Quantification of percentage of tricellular or bicellular cell–cell vertices containing gaps per image (L, M, respectively) from G–K (*n*: 12 images for each genotype, one image from each side from 6 different larvae). Kruskal–Wallis test followed by Dunn’s multiple comparisons, ****P < 0.0001, **P < 0.01, and ns P > 0.05. Confocal images of larval fat bodies expressing Lpp-Gal4+UAS-Myr-td-Tomato +control or UAS-Mys RNAi^27735^ (N, top and bottom, respectively) immunostained for Mys (showing Mys on sides a and b in lateral views). Quantification of mean intensity of Mys for control (N′) and UAS-Mys RNAi^27735^ (N″) on surfaces on sides a and b (mean of mean intensities from several ROIs, data paired by tissue; *n*: six tissues, three surface ROIs per side). Unpaired two-sided *t* test, ****P < 0.0001. **(O–V)** Confocal single Z plane images of larval fat body expressing Lpp-Gal4+UAS-Myr-td-Tomato +control (O), UAS-E-Cad RNAi^103962^ (P), UAS-aPKC RNAi^34332^ (Q), UAS-E-Cad RNAi^103962^+UAS-aPKC RNAi^34332^ (R), UAS-Scribble RNAi^105412^ (S), or UAS-aPKC RNAi^34332^+UAS-Scribble RNAi^105412^ (T; yellow or white arrow showing gaps at tricellular or bicellular vertices, respectively). Quantification of percentage of tricellular or bicellular cell–cell vertices containing gaps per image (U, V, respectively) from O–T (*n*: 12 images for control, E-Cad RNAi^103962^, Scribble RNAi^105412^, and aPKC RNAi^34332^+Scribble RNAi^105412^ one image from each side from six different larvae and 36 images for aPKC RNAi^34332 and^ E-Cad RNAi^103962^+aPKC RNAi^34332^ from six different larvae, half of which from each side). Kruskal–Wallis test followed by Dunn’s multiple comparisons, ****P < 0.0001, ***P < 0.001, **P < 0.01, and ns P > 0.05. Scale bars: 20 µm (A–F, G–K, and O–T).

Our discovery that the larval fat body tissue exhibits apical-basal polarity opened intriguing questions about the functional importance of this polarity. In epithelia, apical-basal polarity proteins are known to regulate cell–cell adhesion via E-cadherin–based adherens junctions (Coopman and Djiane, 2016). To explore the role of apical-basal polarity in the fat body, we next investigated the effects of knocking down the polarity proteins aPKC, Crumbs, Scribble, and Lgl in the fat body. To do this, we first imaged DAPI-stained fat body from wandering third instar larvae expressing UAS-aPKC-RNAi^34332^, UAS-Crumbs-RNAi^39177^, and UAS-Scribble-RNAi^105412^ together with UAS-Myr-td-Tom under the control of an early fat body driver, Lpp-Gal4. Knocking down *aPKC*, c*rumbs*, and *scribbl*e resulted in partial cell–cell dissociation never seen in the control (Fig. 4, A–D, bicellular and tricellular gaps shown with white or yellow arrowheads, respectively). Moreover, knocking down *aPKC* and *scribble* using a second RNAi line (UAS-aPKC-RNAi^105624^ and UAS-Scribble-RNAi^35748^), *crumbs* using two additional RNAi lines (UAS-Crumbs-RNAi^34999^ and UAS-Crumbs-RNAi^330135^), and *lgl* (UAS-Lgl-RNAi^109604^) also resulted in partial dissociation of cells (Fig. S3, A–F), validating our results further.

To quantify the extent of cell–cell dissociation and to assess if these defects are due to aberrant CIVIC-mediated cell–cell adhesion, we next expressed UAS-aPKC-RNAi^34332^, UAS-Crumbs-RNAi^39177^, and UAS-Scribble-RNAi^105412^ with Lpp-Gal4 alongside UAS-Myr-td-Tom and Viking-GFP to quantify CIVIC numbers. We found that aPKC-RNAi^34332^, Crumbs-RNAi^39177^, and Scribble-RNAi^105412^ resulted in 35%, 15%, and 25% of tricellular vertices showing gaps, respectively (Fig. 4, E–I). aPKC-RNAi^34332^ also resulted in 15% of bicellular vertices having gaps (Fig. 4 J). Quantifications of CIVIC numbers on both cell surfaces revealed that the asymmetric localization of CIVICs, with higher numbers seen basolaterally than apicolaterally in the control (Fig. 4, E and E′), as seen before (Fig. 3, F and F′), was disrupted upon aPKC-RNAi^34332^, Crumbs-RNAi^39177^, and Scribble-RNAi^105412^ (Fig. 4, F, F′, G, G′, H, and H′). Strikingly, aPKC and Scribble knockdown resulted in near complete or strong loss of CIVICs on both sides, respectively (Fig. 4, F, F′, H, H′, and K), as well as a reduction in Viking-GFP signal in the BMs (Fig. 4, F″, H″, and L). In contrast, Crumbs knockdown resulted in a redistribution of CIVICs along the lateral domain, with higher numbers of CIVICs on the apicolateral than on the basolateral region (Fig. 4, G, G′, and K). Moreover, it increased the Viking-GFP signal in the BMs (Fig. 4, G″ and L).

Together this showed that aPKC RNAi and Scribble RNAi lead to moderate cell–cell adhesion defects accompanied by defects in CIVIC and BM formation. The extent of the observed adhesion defects, seen mainly at tricellular vertices, was similar to the one found upon strong knockdown of *mys* or *viking* (Fig. S3, G, H, J, and L–N″), key proteins needed for CIVIC-dependent cell–cell adhesion, as shown before (Dai et al., 2017). Next, we assessed whether the remaining cell–cell adhesion seen in these tissues could be mediated by E-cadherin or could be due to incomplete polarity disruption. In contrast to aPKC+Scribble co-depletion (Fig. S3, O, Q, S, T, U, and V), aPKC+E-cadherin co-depletion resulted in a significant increase in cell–cell dissociation at tricellular vertices compared with the single depletions (Fig. S3, O–R, U, and V). Moreover, we saw a similar but nonsignificant increase in dissociation at tricellular vertices for aPKC+Viking co-depletion compared with the single depletions (Fig. S3, G and I–M). This suggests that E-cadherin might mediate some of the remaining cell–cell adhesion observed upon aPKC RNAi.

Together, our results suggest that polarity proteins aPKC, Lgl, Scrib, and Crumbs play an important role in regulating cell–cell adhesion and tissue organization in the larval fat body tissue. aPKC and Scribble, in particular, play a key role in regulating cell–cell adhesion by mediating CIVIC formation.

### aPKC restricts the localization of crumbs, Baz, and Dlg in the fat body

**Figure 5.**
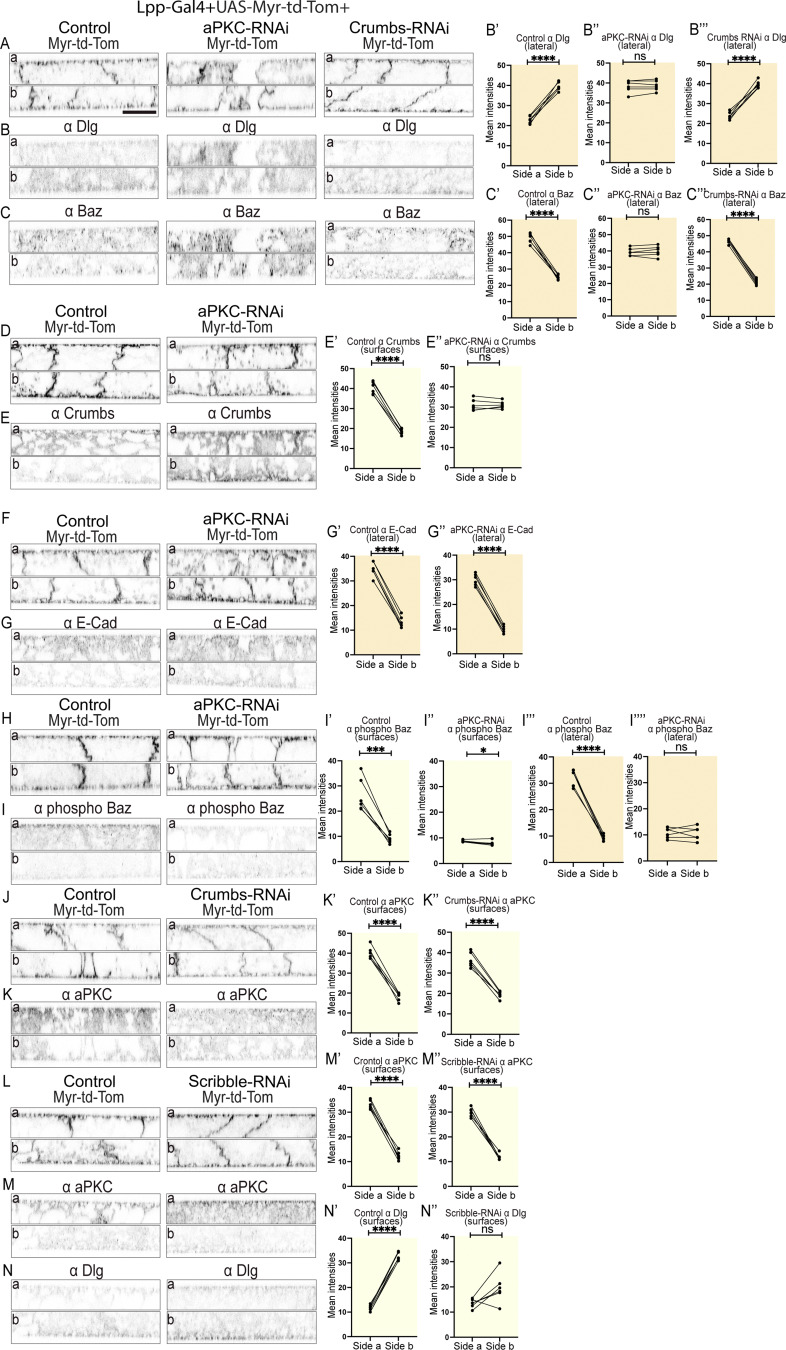
**aPKC restricts the localization of Crumbs, Baz, and Dlg in the fat body. (A–N″)** Confocal images of larval fat body expressing Lpp2-Gal4+UAS-Myr-td-Tom +control, +UAS-aPKC RNAi^34332^, or +UAS-Crumbs RNAi^39117^ (A–C, left, middle and right, respectively) immunostained for Dlg (B) and Baz (C), expressing Lpp2-Gal4+UAS-Myr-td-Tom +control or +UAS-aPKC RNAi^34332^ (D–I, left and right, respectively) immunostained for Crumbs (E), E-Cad (G), or phospho-(PS980)-Baz (I), expressing Lpp2-Gal4+UAS-Myr-td-Tom +control or +UAS-Crumbs RNAi^39117^ (J and K, left and right, respectively) immunostained for aPKC (K) or expressing Lpp2-Gal4+UAS-Myr-td-Tom +control or +UAS-Scribble RNAi^105412^ (L–N, left and right, respectively) immunostained for aPKC (M) and Dlg (N; side a [top] and b [bottom] in lateral view). Quantification of mean intensities of Dlg (B′–B‴, N′ and N″), Baz (C′–C‴), Crumbs (E′ and E″), E-Cad (G′ and G″), phospho-Baz (I′ and I″), and aPKC (K′ and K″, M′ and M″) on surfaces on sides a and b for control (B′, C′, E′, G′, I′, K′, M′, and N′), UAS-aPKC RNAi^34332^ (B″, C″, E″, G″, and I″), UAS-Crumbs RNAi^39117^(B‴, C‴, and K″) or UAS-Scribble RNAi^105412^ (M″ and N″) or on lateral domain for phospho-Baz for control (I‴), UAS-aPKC RNAi^34332^ (I‴′; mean of mean intensities from several ROIs, data paired by tissue; *n*: six tissues, three surface or lateral ROIs per tissue per side). Paired two-sided *t* test, ****P < 0.0001, ***P < 0.001, **P < 0.01, *P < 0.05, and ns P > 0.05. Scale bars: 20 µm (A–N).

Having identified apical-basal polarity in the fat body, we next wanted to investigate how the various cell polarity complexes interact with each other to define the various cortical domains. We found that aPKC RNAi strongly affected the localization of Dlg, Baz, and Crumbs resulting in these proteins now being present at similarly high levels at the apicolateral or basolateral domains or at the apical and basal domains, respectively (Fig. 5, A–B″, C–C″, and D–E′). In contrast, E-Cad localization remained enriched at the apicolateral domain upon aPKC RNAi (Fig. 5, F–G″), in agreement with its potential role in mediating some of the remaining cell–cell adhesion. Using a phospho-specific anti–PS980-Baz antibody, we found that phosphorylated Baz was enriched in the apical as well as the apicolateral domain in control fat body, and this enrichment was lost upon aPKC RNAi (Fig. 5, H–I‴′). We also found that Crumbs RNAi did not alter the basolateral, apicolateral, or apical enrichment of Dlg, Baz, or aPKC, respectively (Fig. 5, A, B, B‴, C, C‴, and J–K″). Finally, Scribble RNAi reduced the basal enrichment of Dlg while not affecting the apical localization of aPKC (Fig. 5, L–N″).

Together, this shows that in the fat body, aPKC acts upstream in the polarity pathway where it regulates the localization of Dlg, Baz and, Crumbs, potentially by restricting their surface distribution. Finally, Baz localization appears to be restricted to the apical and apicolateral domain in part through aPKC-dependent phosphorylation.

### FBCs dissociate during Ecdysone-regulated FBR to initiate amoeboid swimming migration

**Figure 6.**
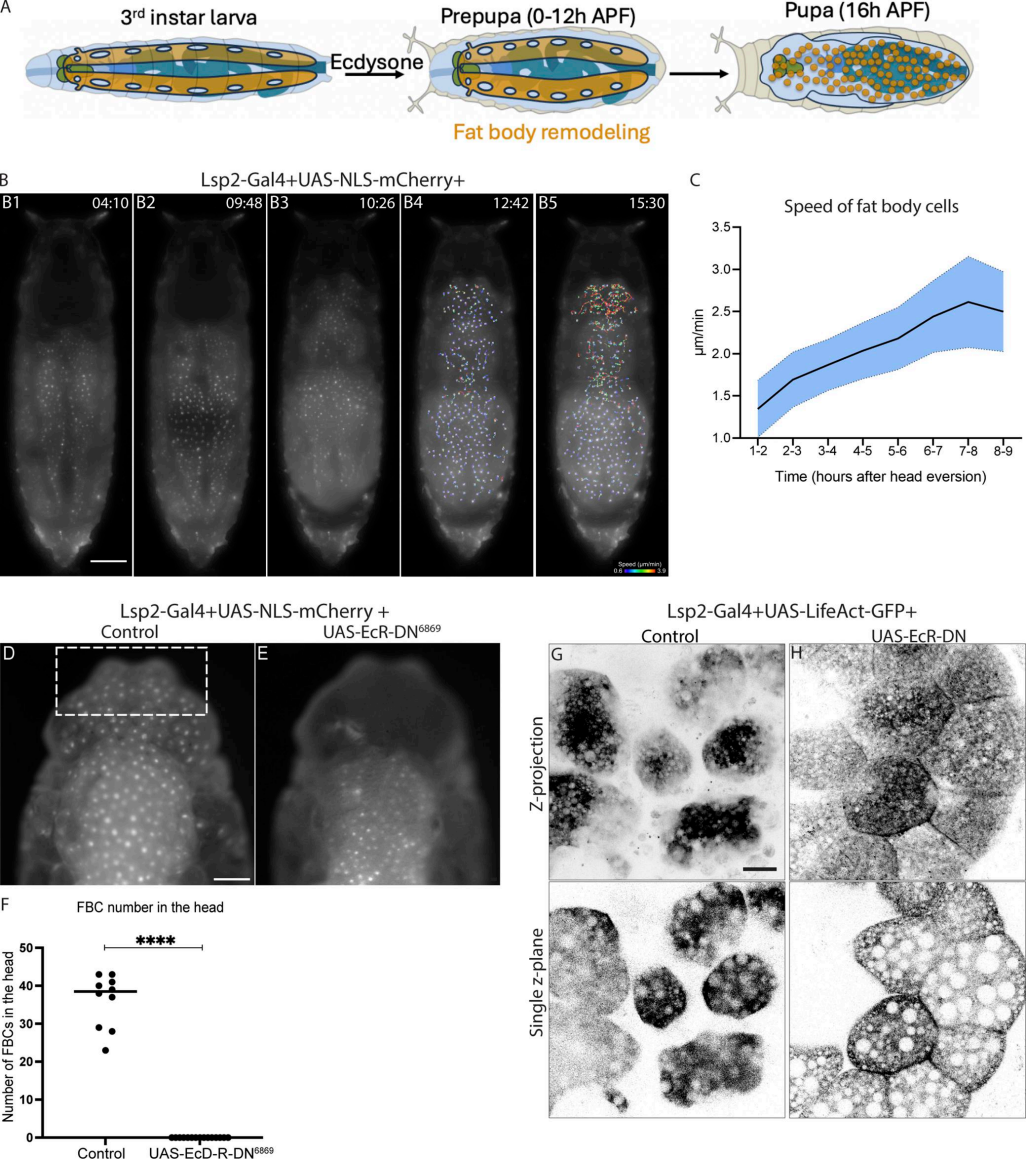
**FBCs dissociate during Ecdysone-regulated FBR to initiate amoeboid swimming cell migration. (A)** Schematic showing fat body morphology before, during, and after FBR (third instar larva, prepupa, and 16 h APF pupa). **(B and C)** Wide-field time-lapse images of the dorsal view of a pupa expressing Lsp2-Gal4+UAS-NLS-mCherry (B1–B5, pupal age 4 h APF at start of movie, imaged at room temperature). Time in hours: min. Migration tracks starting 1 h after head eversion (tracks of minimum length of 90 min): color-coded according to current speed (B4 and B5). Quantification of mean current speed of FBCs in head and thorax over time (C; *n*: 357 tracks from 6 pupae, black line showing mean and standard deviation shown in blue). See Video 1. **(D–F)** Wide-field images of the dorsal view of head and thorax region of 16 h APF pupae expressing Lsp2-Gal4+UAS-NLS-mCherry +control (D) or +UAS-EcRDN^6869^ (E). Quantification of number of FBCs in front of head (F; *n*: 10 pupae [control], 15 pupae [UAS-EcRDN^6869^]; cells counted in front half of head [dotted rectangle in D]). Mann–Whitney test, ****P < 0.0001. **(G and H)** Confocal images of fat body expressing Lsp2-Gal4+UAS-LifeAct-GFP +control (G) and UAS-EcR-DN^6869^ (H, Z projection top and single Z plane bottom). Scale bars: 300 µm (B), 150 µm (D and E), and 20 µm (G and H).

Having established that the larval fat body displays an apical-basal cell polarity that regulates an unusual, CIVIC-mediated cell–cell adhesion mechanism, we next wanted to investigate how cell–cell dissociation during FBR is regulated. FBR happens around 4–14 h APF (Bond et al., 2011; Nelliot et al., 2006) (Fig. 6 A). Having recently discovered that FBCs are migratory in 16 h APF pupae (Franz et al., 2018; Martin et al., 2020), we suspected that FBCs become migratory following FBR. Indeed, we saw this when we imaged FBR *in vivo*. FBCs (nuclei marked) initially remained close to each other within the two lateral sheets and then moved slightly apart from each other (Fig. 6, B1 and B2, respectively, Video 1). Soon after the rear retraction of the animal and head eversion, cells spread across the body (Fig. 6 B3 and Video 1) and started migrating as shown by the gradual increase in cell speed before plateauing 7 h after head eversion (Fig. 6, B4, B5, and C; and Video 1).

**Video 1.** **FBCs dissociate during FBR to initiate cell migration.** Wide-field time-lapse series of the dorsal view of a pupa expressing Lsp2-Gal4+UAS-NLS-mCherry (pupal age 4 h APF at start of movie, imaged at room temperature). Time in hours: min. Migration tracks (shown as dragon tail tracks) starting 1 h after head eversion (only showing tracks of minimum length of 90 min): color-coded according to current speed. See Fig. 6 B.

FBR was strongly blocked when we expressed a dominant-negative version of the Ecdysone receptor (UAS-EcR-DN) together with the nuclear marker UAS-NLS-mCherry using Lsp-Gal4. While FBCs had moved into the head of control pupae after completion of FBR at 16 h APF, the fat body in EcR-DN–expressing pupae remained as sheets in the thorax and abdomen, and no individual cells could be seen in the head (Fig. 6, D–F). Moreover, *in vivo* live imaging of Lsp-Gal4+UAS-Myr-td-Tomato+UAS-EcR-DN–expressing pupae at 16 h APF further showed that the cells remained closely attached in the dorsal abdomen and thorax, while control cells were seen as individual migratory cells (Fig. 6, G and H). This shows that Ecdysone signaling is essential for cell–cell dissociation during FBR, as shown before (Bond et al., 2011; Cherbas et al., 2003).

### Ecdysone regulates cell–cell dissociation through the loss of apical-basal cell polarity and CIVICs during FBR

**Figure 7.**
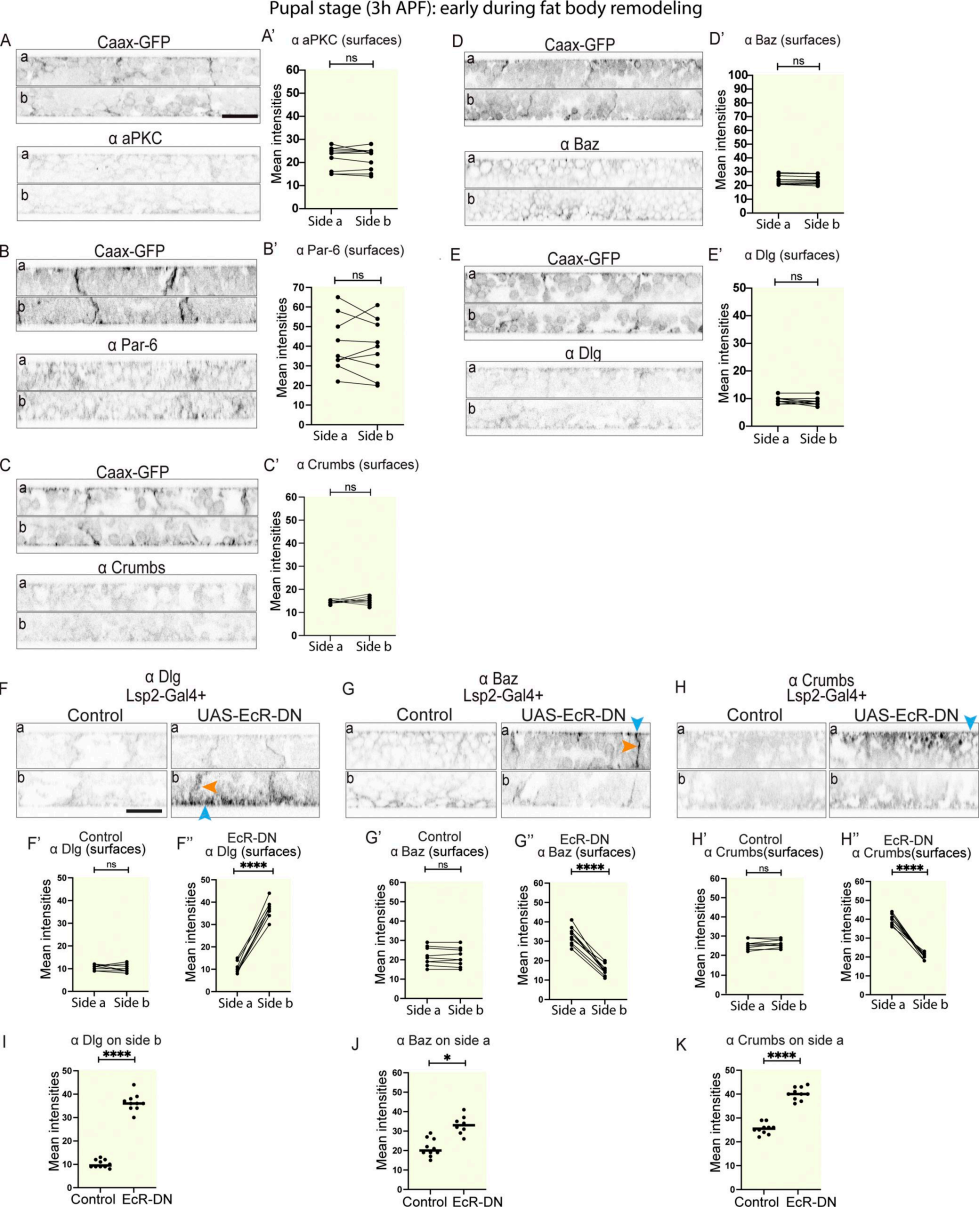
**Ecdysone regulates the loss of apical-basal cell polarity during FBR. (A–E′)** Confocal images of fat body from CAAX-GFP–expressing, 3 h APF pupae immunostained for aPKC (A), Par-6 (B), Crumbs (C), Baz (D), and Dlg (E), imaged on sides a (top) and b (bottom), shown in lateral view. Quantification of mean intensities of aPKC (A′), Par-6 (B′), Crumbs (C′), Baz (D′), and Dlg (E′; at the surfaces, paired for each fat body tissue, *n*: 10 tissues, 3 surface ROIs per tissue per side). Paired two-sided *t* test, ns P > 0.05. **(F–K)** Confocal images of fat body from 3 h APF pupae expressing Lsp2-Gal4 +control or +UAS-EcRDN^6869^ (F, G, and H, left and right, respectively) immunostained for Dlg (F), Baz (G), and Crumbs (H, side a [top] and b [bottom], lateral view, with blue and orange arrowheads pointing at cell surface or lateral domain, respectively). Quantification of mean intensities of Dlg (F′ and F″), Baz (G′ and G″), and Crumbs (H′ and H″) on surfaces on sides a and b for control or UAS-EcRDN^6869^ (mean of mean intensities from several ROIs, data paired by tissue; *n*: 10 tissues, 3 surface ROIs per tissue per side). Paired two-sided *t* test, ****P < 0.0001. Quantification of mean intensities of Dlg (I), Baz (J), and Crumbs (K) at the surface for control and UAS-EcRDN^6869^ on side b (I) or side a (J and K). Unpaired two-sided *t* test, ****P < 0.0001. Scale bars: 20 µm (A–E and F–H).

Having found that apical-basal cell polarity regulates cell–cell adhesion in the larval fat body, we next wanted to see whether apical-basal polarity is lost during FBR before cells dissociate, as during classic EMT. For this, we imaged fat body tissues from 3 h APF-old pupae expressing the membrane marker Ubi-CAAX-GFP immunostained for aPKC, Par-6, Crumbs, Baz, or Dlg. This showed that the asymmetric localization of aPKC, Par-6, Crumbs, and Baz to the apical surface or Dlg to the basal surface that we saw in the larval fat body (Fig. 1, B′–E′ and Fig. 3 A″) was lost at 3 h APF for all these polarity proteins (Fig. 7, A–E′). This suggests that apical-basal cell polarity is lost early during FBR when the cells are still attached to each other.

Next, we wanted to investigate whether Ecdysone signaling regulates this loss of polarity. Immunostaining for Dlg, Baz, and Crumbs showed that in pupae expressing Lsp2-Gal4+UAS-NLS-mCherry+UAS-EcR-DN, Dlg remained concentrated on the basal side, and Baz and Crumbs remained concentrated on the apical side (Fig. 7, F–K), similar to what we saw in larval fat body (Fig. 1, D′ and E′; and Fig. 3 A″), while their asymmetric localization was lost in the control pupae (Fig. 7, F, F′, G, G′, H, and H′). This suggests that Ecdysone signaling in the fat body regulates the loss of apical-basal cell polarity in the fat body early during FBR.

**Figure 8.**
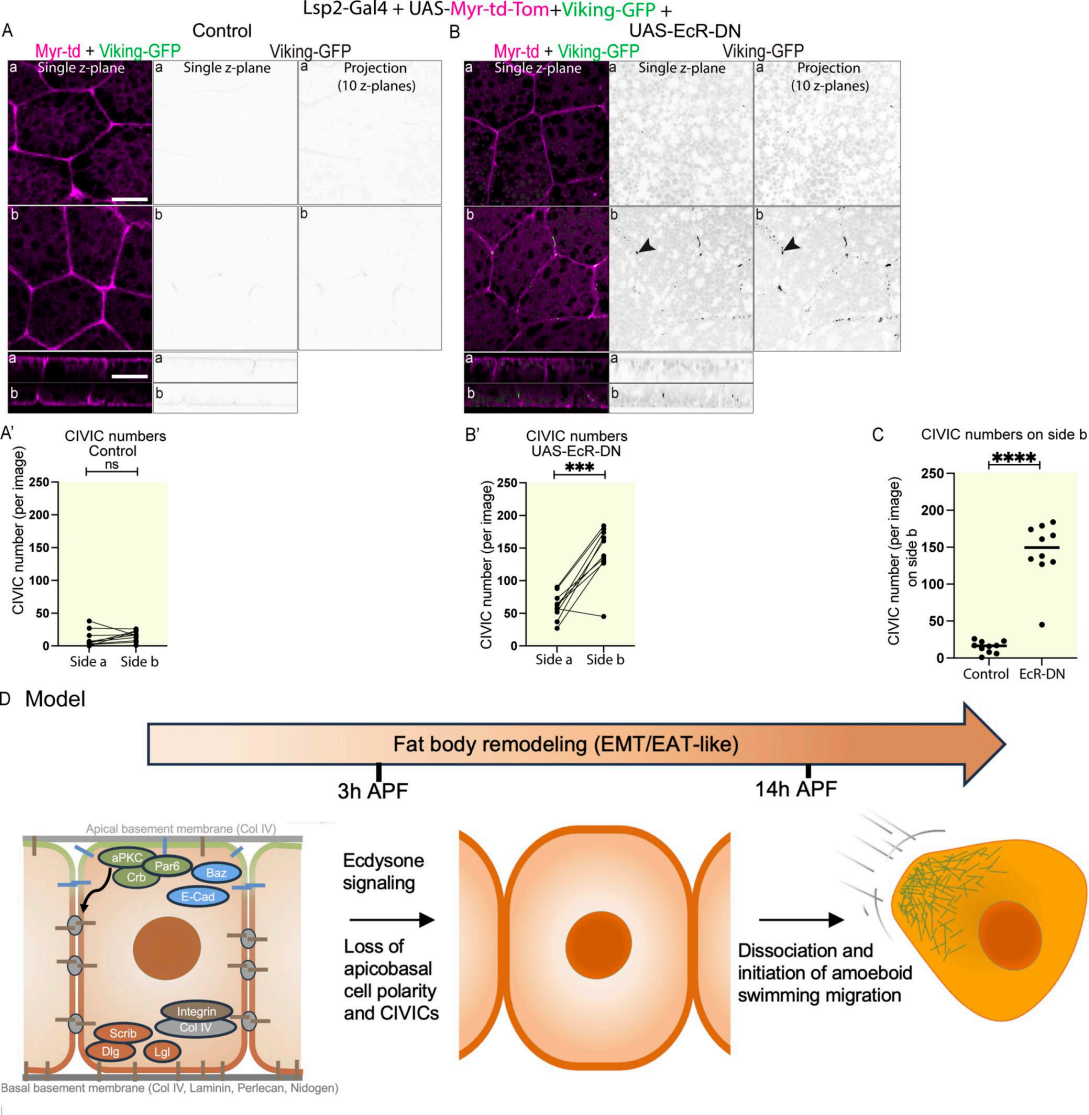
**Ecdysone regulates cell–cell dissociation during FBR through the loss of CIVICs. (A–C)** Confocal images of fat bodies from 3 h APF pupae expressing Lpp-Gal4+UAS-Myr-td-Tomato+Viking-GFP +control (A) or +UAS-EcRDN^6869^ (B; side a and side b shown in planar and lateral view; merge of single Z plane [left] and Viking-GFP channel of single Z plane or of projection of 10 Z planes 2.5–5 μm from cell surface [middle and right panels, respectively]). Quantification of mean Viking-GFP–positive CIVIC numbers (A′, B′, from thresholded Z projection images of 10 Z planes of Viking-GFP channel [2.5–5 μm from cell surface], *n*: 10 tissues, 3 Z projection images per tissue and side, data paired by tissue). Paired two-sided *t* test, ****P < 0.0001 and ***P < 0.001. Quantification of CIVIC numbers for control and UAS-EcRDN^6869^ on side b (C). Unpaired two-sided *t* test, ****P < 0.0001. **(D)** Proposed model of FBR. The fat body in the third instar larva displays an apical-basal cell polarity, which regulates collagen IV–mediated cell–cell adhesion. Ecdysone induces FBR in the prepupa resulting in the loss of apical-basal polarity and CIVICs by 3 h APF. Cells then dissociate and initiate amoeboid swimming migration in pupae around 14 h APF. Scale bars: 20 µm (A and B).

Since cell–cell adhesion in the third instar larval fat body is mediated by CIVICs (Dai et al., 2017), we assessed next whether CIVICs get lost from cell–cell vertices during FBR in WT. Imaging of the fat body from 3 h APF pupae expressing Lpp-Gal4+UAS-Myr-td-Tomato+Viking-GFP showed that CIVIC numbers were very low in the WT fat body (Fig. 8, A and A′), much lower than the numbers seen in the WT fat body of third instar larvae (Fig. 2, F and F′). This shows that CIVICs are lost early during FBR. In contrast, animals expressing UAS-EcR-DN failed to lose CIVICs and had much larger numbers of CIVICs than the control (Fig. 8, B, B′, and C), suggesting that Ecdysone signaling is needed for the loss of CIVICs from the cell–cell vertices early during FBR.

## Discussion

Apical-basal cell polarity is a key hallmark of epithelia, where it dictates their structure and function. One of its key roles is in regulating cell–cell adhesion. Despite being of non-epithelial nature, the mesoderm-derived adipose tissue in flies and humans is composed of tightly associated cells. The single-layered fat body in *Drosophila* is ensheathed in a BM. In mammals, adipocytes are found in various multilayered adipose tissue depots in which the cells are each surrounded by a thick collagen IV–containing ECM that provides mechanical support (Mariman and Wang, 2010). The mechanisms that regulate adipose tissue architecture as well as the functional significance of this architecture are still mostly unknown. Here we show that the adipose tissue in flies, the fat body, previously not thought to be polarized, in fact displays an apical-basal cell polarity, which is essential for cell–cell adhesion and tissue integrity (Fig. 8 D). Strikingly, in contrast to epithelia, the apical-basal polarity machinery in the fat body regulates cell–cell adhesion primarily via collagen IV. An interesting question that arises from this discovery is what the underlying mechanism may be. Apical-basal polarity proteins usually determine the apical, apicolateral, basolateral, and basal cell domains. These, in turn, might regulate the architecture of the cytoskeleton, which could direct localized secretion of Col IV and/or transport of integrin to specific sites in the lateral domain to initiate CIVIC formation. However, the fat body has a centrally positioned perinuclear MTOC from which microtubules grow radially toward the cell periphery (Sun et al., 2019; Zheng et al., 2020) and kinesin RNAi does not affect CIVIC formation (Zheng et al., 2020). Interestingly, Col IV fibrils, which are of similar composition and structure to CIVICs, have been described to form at cell–cell interphases in *Drosophila* follicle cells, a type of somatic epithelial cell (Isabella and Horne-Badovinac, 2016). These Col IV fibrils form through directed Rab10-mediated secretion of Col IV into the pericellular space at the basal region of cell–cell interfaces, from where they are subsequently incorporated into the underlying BM (Isabella and Horne-Badovinac, 2016). A similar mechanism of directed Col IV transport into the lateral plasma membrane might also mediate CIVIC formation in the fat body.

Col IV–dependent cell–cell adhesion has so far only been reported for the fat body, but it might not be unique to this tissue. As mentioned above, Col IV fibrils that form in the pericellular space in follicle cells have a similar localization and ECM composition to CIVICs (Isabella and Horne-Badovinac, 2016) and might hence be related structures. While these Col IV fibrils have not been reported to mediate cell–cell adhesion, it is tempting to speculate that they could contribute to cell–cell adhesion in the basolateral cell region of follicle cells before being deposited into the BM. Moreover, there are also some known examples where integrin-binding to ECM can mediate cell–cell adhesion. For example, integrins at myotendinous junctions are known to connect muscle cells to tendon cells through an intervening ECM (Maartens and Brown, 2015).

It may be that the use of CIVICs for cell–cell adhesion is unique to tissues that secrete collagen IV, like the fat body. Interestingly, there are two populations of Col IV that FBCs (Dai et al., 2017) and follicle cells (Isabella and Horne-Badovinac, 2016) produce, one secreted to the surface to form the BM and one secreted laterally to form Col IV concentrations in the pericellular space. Hence, it might be that CIVIC-dependent cell–cell adhesion and focal adhesion-mediated cell–BM adhesion are part of an interlinked mechanism that has coexisted during evolution. Early in evolution apical-basal polarity might have regulated Integrin-binding to locally secreted Col IV-containing ECM, which in turn, could have mediated cell–cell adhesion while simultaneously mediating cell–BM adhesion. This could hence constitute an ancient mechanism enabling the evolution of multicellular animals from their unicellular ancestors. Both the integrin adhesion machinery (Sebe-Pedros et al., 2010) and the E-cadherin adhesion machinery (Murray and Zaidel-Bar, 2014) have been reported to have an ancient origin predating the emergence of metazoans and might hence have evolved in parallel. In most tissues in extant animals, E-cadherin–mediated adhesion might then have taken a dominant role over Col IV–dependent cell–cell adhesion.

Our study reveals that the fat body has some features of classic epithelia, including the presence of an apical-basal cell polarity machinery. Moreover, aPKC-dependent phosphorylation of Baz in the fat body appears to mediate the apicolateral localization of Baz even though it does cause strong apical exclusion like in epithelia (Morais-de-Sa et al., 2010; Walther and Pichaud, 2010). However, there are also some key differences between epithelia and the fat body. We find that aPKC RNAi in the fat body results in Crumbs being present at similarly high levels at the apical and basal domains. In contrast, the cell surface localization of Crumbs is lost in *apkc* mutant epithelia (Harris and Peifer, 2005; Morais-de-Sa et al., 2010; Walther and Pichaud, 2010). The remaining high localization of Crumbs in the apical domain upon aPKC RNAi in in the fat body could be due to Crumbs binding to Stardust or due to incomplete depletion of aPKC. However, this would not explain the aberrantly high basal localization of Crumbs that we also see. Therefore, this rather suggests that the mechanism that mediates the trafficking of polarity proteins to the cell surface might be different in the fat body than in epithelia. Crumbs recruitment to the cell surface in the fat body might not require aPKC. Instead aPKC might restrict the localization of these apical polarity proteins to certain domains following surface recruitment. It would be interesting to study the trafficking of polarity proteins in the fat body further to see if the underlying mechanism is linked to the unusual trafficking of ECM proteins that also appears to occur in this tissue.

Another key difference that our study finds between epithelia and the fat body that our study finds regards the BM. Epithelia have a Col IV–containing BM only on the basal side, while they also often have an apical ECM devoid of Col IV. In contrast, we find that the fat body has two distinct BMs, one containing abundant levels of Col IV, Laminin, Perlecan, and Nidogen on the basal surface, and one mainly containing Col IV on the apical surface. Interestingly, we find integrin, which mediates cell–BM binding (Hynes and Naba, 2012; Leitinger, 2011; Yamada and Sekiguchi, 2015), is enriched basally and is present at lower levels apically. This suggests that that the ECM levels are proportional to the levels of integrin in these two BMs. Further research is needed to reach a better molecular understanding of how apical-basal cell polarity in the fat body regulates the formation of these distinct apical and basal BMs.

What is the function of apical-basal cell polarity in the larval fat body? Our results show that the apical-basal polarity proteins aPKC, Crumbs, Lgl, and Scribble are required for intercellular adhesion of FBCs. aPKC and Scribble, in particular, play a key role here in mediating cell–cell adhesion by regulating CIVIC formation. Apart from this, it seems likely that apical-basal cell polarity also plays other roles in regulating cell function. It is tempting to speculate that some of the well-established functions of the fat body, including lipid uptake or release (Arrese and Soulages, 2010), antimicrobial peptide secretion to fight infection (Ferrandon et al., 2007) or tumors (Parvy et al., 2019), or secretion of other factors such as growth factors (Agrawal et al., 2016; Géminard et al., 2009; Sousa-Nunes et al., 2011), might be mainly mediated via either the apical or the basal cell surface.

In addition to providing new insight into how adipose tissue architecture is regulated, our study also sheds new light on the process of FBR. We show Ecdysone signaling in the fat body induces cell–cell dissociation by regulating the loss of apical-basal cell polarity and CIVICs (Fig. 8 D). Interestingly, our new findings, together with our previous findings (Andrieu et al., 2025), show that FBR is followed by initiation of amoeboid swimming migration. FBR and EMT have several key features in common. First, in both cases there is an apical-basal cell polarity mediating cell–cell adhesion, albeit through different mechanisms, which is then lost during the process to induce cell–cell dissociation. Second, matrix metalloproteinases induce loss of cell–BM adhesion (Bond et al., 2011; Jia et al., 2014; Lamouille et al., 2014). Third, at the end of the process, in both cases cells become motile albeit using different modes of migration. FBCs use amoeboid swimming cell migration (Andrieu et al., 2025), whereas most cells undergoing EMT that have been studied so far use mesenchymal cell migration. However, some cancer cells undergo an epithelial-to-amoeboid transition, resulting in amoeboid cell migration (Graziani et al., 2022). Interestingly, during wound healing in mice, adipocytes have been shown to become migratory to invade the wound bed, suggesting that they might also undergo an EMT-like process (Kalgudde Gopal et al., 2023; Shook et al., 2020). It remains to be seen whether the adipose tissue in mammals might also display an apical-basal polarity.

Taking all of this into account, we propose that the remodeling of the *Drosophila* adipose tissue constitutes a novel category on the spectrum of EMT/EAT. This powerful genetic *in vivo* model system could be a valuable addition to the small set of commonly used EMT models, which could be helpful for unraveling the diverse mechanisms underlying EMT and EAT in health and disease.

## Materials and methods

### Fly stocks and maintenance

*Drosophila melanogaster* stocks and crosses were maintained and performed on cornmeal molasses food at 25°C. Stocks obtained from the Bloomington *Drosophila* Stock Center (NIH P40OD018537) were used in this study. The following lines were used in this paper: w^67^ or UAS-NLS-LacZ (BDSC: 3956) as a control; Lsp2-Gal4 (BDSC: 6357); Lpp-Gal4 (gift from Pierre Leopold, Institut Curie, Paris, France); Ubi-CAAX-GFP (DGRC: 109824); UAS-EcR-B1-DN (BDSC:6869); UAS-LifeAct-GFP (Zanet et al., 2012); UAS-Myr-td-Tom (BDSC: 32221); UAS-NLS-mCherry (BDSC: 38424); Lgl-GFP (BDSC: 63183); UAS-aPKC-RNAi 1 (BDSC: 34332) and 2 (BDSC:105624); UAS-Scribble-RNAi 1 (BDSC: 35748) and 2 (BDSC: 105412); UAS-Crumbs-RNAi 1 (BDSC: 34999), 2 (VDRC: GD-39177), 3 (VDRC: shRNA-330135), and 4 (BDSC: 40869); UAS-Lgl-RNAi (VDRC: KK-109604), UAS-ECad-RNAi (VDRC: KK-103962), 2 (VDRC: GD-27082), and 3 (BDSC: 32904); UAS-Mys RNAi (BDSC: 27735); UAS-Viking RNAi (VDRC: 106812); UASp-GFP-Golgi (BDSC:31422); Laminin B1-GFP (Sarov et al., 2016); Nidogen-GFP (Sarov et al., 2016); Trol-GFP (110836; Kyoto Stock Centre); Viking-GFP (DGRC: 110626); Venus-Ilk (gift from Nic Brown, Department of Physiology, Development and Neuroscience, Cambridge University, Cambridge, UK); If-YFP (115467; Kyoto Stock Centre); and Dystroglycan-GFP (Villedieu et al., 2023). We used FlyBase to find information on phenotypes/function/stocks/gene expression (etc.).

### Fat body dissections

Wandering third instar larvae were dissected on a sylgard-coated depression dish. Animals were placed on their dorsal side and pinned by the tail and mouth hooks. Using spring-scissors, a horizontal incision was made in the posterior end of the larva, followed by a vertical cut along the dorsal midline toward the rostral end of the larva. Then a horizontal cut was made left and right of the pin at the rostrum of the animal. The flaps were then pinned in a clockwise order to ensure that the animal’s body was stretched both horizontally and vertically. The animal was fixed with 4% paraformaldehyde for 30 min, to allow organs to float and facilitate organ removal, including fat body tissues. For fat body dissections, the trachea along with the gut were first removed, ensuring the fat bodies were kept along the sides of the animal. An incision on the anterior end of the right side of the fat body was then made. The right halves of fat body tissues were then placed in 96-well plates and washed twice with PBS. The dissected tissues were sometimes stored in PBS for up to 5 days at 4°C. This dissection method was also used to dissect fat body tissues from 3 h APF pupae.

### Immunohistochemistry

Dissected fat body tissues were fixed in PBS containing 4% paraformaldehyde for 30 min, permeabilized in PBS containing 1% Triton X-100 at room temperature, and blocked in PBT with 4% fetal bovine serum. The dissected tissues were incubated overnight at 4°C with Phalloidin-CF488A (00042-T; Biotium) or primary antibodies mouse anti-Crb (1:50; DSHB AB_528181 [cq4]), mouse anti-Dlg1 (1:20; BSHB AB_528203 [4f3]), rabbit anti-Baz (1:2,000; a gift from Andreas Wodarz, University of Cologne, Köln, Germany), rabbit anti-PS980-Baz (1:200 [Morais-de-Sa et al., 2010]), guinea pig anti-Par-6 (1:500 [Walther et al., 2016]), rabbit anti-aPKC (1:500; SAB4502380; Sigma-Aldrich), rat anti-DE-Cadherin (1:20; DSHB AB_528120 [E-CAD2]), and mouse anti-Mys (1:20; DSHB AB_528310 [cf.6g11]) diluted in PBT. After three washes in PBS, the dissected tissues were incubated with secondary antibody, anti-rat Alexa Fluor 568 (1:200; A-11077; Thermo Fisher Scientific), anti-mouse Alexa Fluor 647 (1:200; A-21241; Thermo Fisher Scientific), anti-guinea pig Alexa 488 (1:200; A-11073), and anti-rabbit Alexa Fluor 488 (1:200; A-11011; Thermo Fisher Scientific) for 2 h at room temperature. Fixed and stained samples were mounted on DAPI-vectashield (S36973; Thermo Fisher Scientific) and prepared for imaging.

### Microscopy

#### Imaging setup to image dissected fat body

Stained right halves of fat body tissues dissected from different animals were mounted between two coverslips. Side (a) and side (b) of the right half of the fat body could be identified the following way: The fat body sheet has several round gaps in the tissue. The tissue is wider (∼4–7 FBCs wide) on one side of the gaps than on the other side (∼1–2 FBCs wide; see Fig. 1 A). For imaging side (a), the fat body sheet was oriented with the anterior end upward and the posterior end downward, ensuring that the thicker side was pointing left and the thinner side right. On image side (b), the cover glass was flipped over so that the anterior end pointed upward and the posterior end downward, and the thinner side was located to the left and the thicker side to the right.

#### Imaging setup to image dissected pupae

For imaging done on pupae, animals were kept at 25°C. Pupae were marked at the white prepupa stage (0 h AFP) and dissected at 16 h APF by removing the pupal case (Weavers et al., 2018) and placed on a coverslip on their dorsal side for imaging.

#### Microscopy

Microscope images were collected at room temperature on a Zeiss 980 Airyscan 2 inverted point scanning confocal microscope using a Plan-Apochromat 63 × 1.40 NA oil objective at 0.25 μm step size with a Zen blue acquisition software except for Fig. 6, B, D and, E; and Video 1, which were collected on a Zeiss Cell discoverer CD7 inverted wide-field microscope with a 5× air objective with a 0.5 optovar to give 0.5× magnification and a sCMOS camera and with a Zen blue acquisition software. Video 1 was acquired for 19 h 24 min with a time interval of 2 min at room temperature. Nuclear tracking in Video 1 was done as described before (Andrieu et al., 2025) as follows. Nuclear tracking was performed automatically in an unsupervised manner using Imaris software (Oxford Instruments) in the dorsal head and thorax of pupae. The tracking was obtained in 2D using *Z* projections of the 3D movies. Tracking was done every 2 min. Only tracks with a duration of a minimum of 90 min were selected and analyzed. We then examined visually to see whether the tracks were correct. Incorrect tracks were either corrected or deleted. Average migration speed per pupa was obtained by averaging the mean speed of all individual tracks at various 1 h time intervals (e.g., 1–2, 2–3 h etc. after head eversion). Tracks (of a minimum tracking length of 90 min) were color-coded based on their current speed over time in Imaris. Tracks are shown in images as dragon tail tracks.

Images and movies of confocal movies were generated with Fiji ImageJ to create Z-projections, Z-sections, and orthogonal view images. We used the same brightness and contrast adjustment for control and experimental conditions. Movies and images were organized and annotated with VSDC Video Editor and Abode Illustrator.

### Electron microscopy

Dissected fat body tissues from wandering third instar stage larvae were fixed in 2% paraformaldehyde and 1.5% (vol/vol) glutaraldehyde in 0.1 M cacodylate. Following 0.1 M cacodylate washes, fat body tissues were fixed in 1% osmium tetroxide and 1.5% K3[Fe(CN6)] for 1 h at room temperature and rinsed with ddH_2_O. After osmium-ferricyanide staining, the tissues were treated with 1% thiocarbohydrazide for 20 min at room temperature, stained with 2% osmium tetroxide for 30 min at 4°C, washed three times with ddH_2_O, and stained with 1% aqueous uranyl acetate overnight at 4°C. Next, the tissues were incubated with freshly made lead aspartate solution for 30 min at 60°C. After being rinsed with buffer and gradually dehydrated with increasing concentrations of ethanol (70, 90, and 100%), the tissues were infiltrated with a graded series of EPON. The tissues were then placed in blocks, polymerized in 100% resin, and baked overnight at 60°C. Electron microscopy was performed with a Tecnai G2 Spirit transmission electron microscope (FEI) equipped with a Morada charge-coupled device camera (Olympus Soft Imaging Systems).

### Image analysis

#### Nuclear positioning

The distance between the nuclear surface and the cell surface was measured manually in FIJI-ImageJ by counting the number of z slices from the cell surface to the onset of nuclear DAPI signal.

#### CIVIC numbers

CIVICs quantifications were performed on the maximum projection of 10 layers within the Z-stack (2.5–5 μm from the cell surface). A threshold rage of 43–255 was applied in Fiji, and particles with sizes from 0.2 to infinity (pixel^^^2) were identified and analyzed.

#### Analysis of bicellular or tricellular gaps

The number of bicellular or tricellular cell–cell vertices with or without gaps was counted manually using the cell counter plugin on FIJI-ImageJ at 7.5–20 μm from cell surface to calculate the percentage of vertices containing gaps.

#### Mean intensity analysis at cell surfaces and lateral sides

Mean fluorescence intensities were measured using ROIs (one for surface measurements [46.69 × 3.29 μm [wide/high]), shown in yellow in Fig. 1 A and one for lateral measurements (3.62 × 4.60 μm [wide/high]), shown in orange in Fig. 1 A) or ROIs of 43.93 × 3.95 μm (wide/high) for measurements of actin and Golgi in the region under the cell surface. The mean of the mean intensities of several regions imaged on the same side (either side a or b) of the same fat body tissue was calculated for each fat body tissue and shown paired per tissue in the graphs.

#### FBC counts in pupal head

FBCs in the head of pupae (Fig. 6, D and E) were counted manually in the front half of the head of the pupae using the cell counter plugin on FIJI-ImageJ.

### Statistics

Statistics were performed using GraphPad Prism 9. Statistical tests used in each experiment are indicated in the relevant figure legends. P < 0.05 was set as the significance threshold. In scatter dot plots, the line in the middle indicates the median.

### Online supplemental material

Fig. S1 shows lateral view of FBCs imaged across the whole tissue, quantifications of CAAX-GFP on both surfaces (related to Fig. 1), quantification of actin, the Golgi apparatus, nuclear positioning, and Ilk and If. Fig. S2 shows E-Cad RNAi in fat body using two additional RNAi lines (related to Fig. 3 C). Fig. S3 shows RNAi of aPKC, Crumbs, Scribble, and Lgl using additional RNAi lines (related to Fig. 4 A) and double knockdown of aPKC+E-Cad and aPKC+Scribble as well as quantifications of gaps in tricellular and bicellular vertices. Video 1 shows nuclear behavior of FBCs during FBR.

## Data Availability

All data associated with this study are present in the paper or the supplemental information. This paper does not report any code or informatics dataset. Any additional information required to reanalyze the data reported in this paper is available from the lead contact upon reasonable request.
